# Applying a Combination of SEM and fsQCA to Predict Tourist Resource-Saving Behavioral Intentions in Rural Tourism: An Extension of the Theory of Planned Behavior

**DOI:** 10.3390/ijerph20021349

**Published:** 2023-01-11

**Authors:** Wei Zheng, Hongliang Qiu, Alastair M. Morrison

**Affiliations:** 1 School of Tourism and Foreign Languages, Tourism College of Zhejiang, Hangzhou 311231, China; 2 Postdoctoral Station of Business Administration, Fudan University, Shanghai 200433, China; 3 School of Business Administration, Tourism College of Zhejiang, Hangzhou 311231, China; 4 Zhejiang Academy of Culture & Tourism Development, Hangzhou 311231, China; 5 School of Management and Marketing, Greenwich Business School, University of Greenwich, Old Royal Naval College, Park Row, London SE10 9LS, UK

**Keywords:** tourist resource-saving behavioral intentions, theory of planned behavior, fuzzy-set qualitative comparative analysis (fsQCA), environmental concern

## Abstract

How to save resources and protect the environment at destinations is one of the hot issues in tourism. One effective solution is to cultivate tourist resource-saving behavioral intentions (TRSBI). Prior studies mainly use Structural equation modeling (SEM) to explore its antecedents, whereas other potential methods (i.e., fuzzy-set qualitative comparative analysis, fsQCA for short) have been less adopted. This study combines SEM and fsQCA to examine TRSBI in a rural tourism context. Specifically, SEM is executed to investigate how environmental concern influences TRSBI based on the theory of planned behavior (TPB), while fsQCA is applied to uncover the multiple configurations in the TRSBI formation. The findings from SEM indicated that (1) environmental concern positively and directly influenced TRSBI; (2) TPB constructs (i.e., attitudes toward the behavior, subjective norms, and perceived behavioral control) positively and separately mediated the associations of environmental concern with TRSBI. The fsQCA outcomes showed that three configurations result in a high level of TRSBI: (1) high attitudes toward the behavior, subjective norms, and perceived behavioral control, (2) high attitudes toward the behavior, subjective norms, and environmental concern, and (3) high attitudes toward the behavior, perceived behavioral control, and environmental concern. The combined approaches offer a systematic and holistic solution to explore TRSBI in rural tourism.

## 1. Introduction

Rural tourism develops at a rapid pace worldwide [1]. Enjoying advantages of natural landscape, traditional culture, and leisure traits, rural tourism plays an irreplaceable role in tourism sector, and makes significant contributions to economic revitalization, poverty alleviation, agricultural transformation, natural resources management, local culture revival, and resident well-being improvement [2,3]. It has also been particularly favored by people during COVID-19 pandemic [4]. Having been restricted from international travels, people are cautious about population density, space, and restorative environment [5]. Working as an effective development strategy, rural tourism attracts increasing attention from both scholars and practitioners with the focus on sustainability [6].

Tourists’ deep engagement in tourism inevitably brings negative environmental effects at destinations [7]. Rural destinations are challenged with overcrowding, increased greenhouse gas emissions, water pollution, vegetation destruction, mounting waste, and resources overuse [8,9]. Ecological environment and resources have laid foundation for tourism development, but public unawareness of and indifferent regard for them greatly weaken a destination’s appeal [10]. It is particularly true with rural destinations. Resources, especially natural resources, constitute rustic landscapes which are at the core of rural destination attractiveness [11]. The #15 Sustainable Development Goal (SDG) of the 2030 Agenda for Sustainable Development calls on people worldwide to “protect, restore, and promote sustainable use of terrestrial ecosystems, sustainably manage forests, combat desertification, and halt and reverse land degradation and halt biodiversity loss” [12,13]. As such, resources at rural destinations should be well preserved and sustained. Environmental issues could be managed through correcting individual behaviors [14], and behavioral changes can lead to significant reduction of resource consumption [15]. Tourists are crucial stakeholders at destinations [16], and resource-saving behavior as a key factor mitigates negative environmental effects caused by tourism activities [17]. To ensure sustainability in rural areas, it is imperative to nurture tourist resource-saving behavioral intentions.

Regarding the research on resource-saving behavior, a plethora of literature has centered on its segmentation including water-saving and energy-saving behaviors, squarely with household, workplace, and hotel settings [17,18], lacking a comprehensive perspective and broader context. To fill the gap, a theoretical framework is of prime importance. Among the most frequently used theories for predicting human behaviors, the theory of planned behavior (TPB) is one of the dominant paradigms [19]. TPB holds that one’s intention to perform a certain behavior is a proxy for actual behavior [20,21], while this intention is driven by the three endogenous components, i.e., attitudes toward the behavior, subjective norms, and perceived behavioral control [22]. TPB has well predicted pro-environmental behaviors and intentions, including ecological behavior, environmental behavior, green hotel consumption choice, pro-environmental behavior and low-carbon travel intentions [23]. Resource-saving behavior as an important segment of pro-environmental behavior is expected to justify the robustness of TPB. In addition, as TPB is centered on rational attributes of individual behaviors, it is acknowledged that TPB’s extension with constructs included according to specific situations is necessary and could enhance its explanatory power [24,25]. Hence, there are solid grounds for this research to apply the TPB framework to explore tourist resource-saving behavioral intentions.

Rural tourism development leans on natural resources. Influential factors guiding tourists to save resources could include tourist concern for the environment [26,27]. A widespread notion of environmental concern is that it is fundamental in environmental research, a significant determinant during consumer decision-making process [28] and a valuable driver of pro-environmental behavior [29]. In rural settings, rustic environment, nature-based activities, and local green food rely on and develop under green principles. For instance, nature-based activities encourage tourists to get more connected to nature by minimizing automobiles and enjoy walking, bicycles, battery carts, and local transportation, since speed and carbon emission destroy the connection with rural resources; meanwhile relate their environmental concern to polices including landscape sustainability, air and water quality supervision, waste management, pollution control, resource saving, and alternative energy application [30,31]. Therefore, rural tourists are more sensitive to environmental problems, more environmentally concerned, and more appreciative of resource-saving behavior. A stronger environmental concern is more likely to provoke willingness to pro-environmental engagement [32,33]. Unfortunately, the relationship between environmental concern and tourist resource-saving behavior has been less discussed with regard to rural tourism in the extant literature. As such, it provides an opportunity for this TPB research to include environmental concern and examine its effect on tourist resource-saving behavioral intentions.

In addition, environmental concern is ascribed to general attitudes formed toward real-world phenomena, while attitudes toward the behavior as a TPB endogenous construct tend to demonstrate specifically positive/negative evaluation toward a certain object [34]. Studies demonstrated that environmental concern (i.e., general attitudes) positively affected specific attitudes, and further impacted individual decision making [30]. Undoubtedly, environmental concern can be treated as a separate antecedent beside attitudes in this research. Regarding the underlying differences resulted by influences of two types of attitudes on individual behavior, it is therefore important to investigate the influence mechanism of tourist resource-saving behavioral intentions by integrating both attitudes (i.e., environmental concern and specific attitudes toward the behavior).

To identify and compare configurations leading to resource-saving behavioral intentions, fuzzy-set qualitative comparative analysis (fsQCA) was applied in this research. The reasons are threefold. First, a traditional analysis method like Structural equation modeling (SEM) is good at examining variables’ linear connections and emphasizing simple and symmetric associations between antecedents and outcomes, but is unable to demonstrate complex multi-factorial and concurrent causality; second, fsQCA facilitates the examination of aggregation relationships and further comprehensive implementation paths; third, given that social phenomenon is a complex and its causal mechanism alters due to various conditions, it is necessary to systematically identify causal logics so as to better explain complicated social phenomenon [35]. The combination of SEM and fsQCA has gained positive results in tourism management [36,37,38]. To this end, the current study adopted both SEM and fsQCA as a more comprehensive methodological perspective to understand the formation mechanism of tourist resource-saving behavioral intentions in rural tourism context.

Given the above argument, the current study applied the theory of planned behavior (TPB) as the theoretical underpinning for exploring the formation of tourist resource-saving behavioral intentions. The objectives of this research were to: (1) analyze tourist resource-saving behavioral intentions in rural tourism context and employ the theory of planned behavior to fulfill it; (2) test the mediating role of TPB constructs (i.e., attitudes toward the behavior, subjective norms, and perceived behavioral control) in the relationship between environmental concern and tourist resource-saving behavioral intentions; and (3) reveal the causal configurations that lead to tourist resource-saving behavioral intentions and apply fsQCA to achieve it. This research enriches our understanding of tourist resource-saving behavioral intentions from a methodological perspective. The extended TPB model with the combination of SEM and fsQCA allows for a more comprehensive way to exploring the mechanism forming tourist resource-saving behavioral intentions in rural tourism contexts, and offers a practical reference for destination management organizations by strengthening the synergistic integration of attitudes toward the behavior, subjective norms, and perceived behavioral control as well as environmental concern and attitudes toward the behavior to promote resource-saving behavioral intentions.

## 2. Literature Review and Hypotheses Development

### 2.1. Tourist Resource-Saving Behavioral Intentions (TRSBI)

Resource saving nowadays does not mean reducing food and clothing, nor simply suppressing reasonable consumption needs. It has a brand-new decipherment: to make sustainable management of resources so as to ensure social sustainable development. As such, resource-saving behavior could be interpreted as reduction, reuse, and recycling behaviors that people perform in daily life motivated by resource sustainable use and social sustainable development goals [39].

Resource-saving behavior is among main ways to strengthen resource efficiency and helps mitigate environmental problems including climate change, natural resource diminution, and waste issues [17]. Therefore, it is prone to being altruistic in nature. Its segment behaviors include purchase, usage, and disposal, some of which feature immediate (direct) resource consumption reduction (e.g., reuse, water and electricity conservation, and plastic bag reduction), and some of which stress long-term (indirect) efforts (e.g., purchasing energy-saving and high-efficient household appliances, and selecting new energy vehicles) [39]. The research hereby refers tourist resource-saving behavioral intentions to tourist willingness to conduct altruistic behaviors on reduction, reuse, and recycling in order to promote resource efficiency and sustainability at tourism destinations.

### 2.2. Theory of Planned Behavior (TPB) and the Extension of TPB

The theory of planned behavior is regarded as one of the most effective theoretical frameworks in predicting and examining human behaviors [12], including a wide range of pro-environmental behaviors [40]. TPB has been applied to examine segment behaviors and intentions including energy saving [41], electricity saving [42], water saving [43], food waste reduction [44], e-waste management [45], recycling [46], reuse [47], sustainable purchase [48], renewable energy technology installation [49], transport mode shift e.g., using and sharing bicycles [34,50], and adopting hybrid electric vehicles [51] and banning gasoline ones [52]. However, resource-saving behavior as a whole has not yet been studied. Backed by the above evidence, this research used TPB as a fundamental and feasible theoretical framework to explore tourist resource-saving behavioral intentions.

Three endogenous components constitute TPB, all of which lead to individual behaviors [22]. The first is attitudes toward the behavior which are defined as a person’s overall appraisal of performing a behavior [22]. Attitudes represent a person’s psychological assessment procedure before performing an action [12]. According to the expectation–disconfirmation paradigm, attitudes exhibit causal nexus with behavior, i.e., a favorable expectation evokes positive attitudes which further produce a positive motivational force to provoke behavioral intentions, while an unfavorable expectation elicits negative attitudes that lead to behavior avoidance [53]. For instance, attitudes have been found to exert a significant influence on both household and workplace energy saving intentions [54,55]. Given the above discussion, the research’s first hypothesis was:

**H1.**  *Attitudes toward the behavior positively impact tourist resource-saving behavioral intentions.*

The second component is subjective norms which are conceptualized as perceived social pressure when an individual is considering whether or not to conduct a behavior [22]. Subjective norms emphasize that an individual tends to listen to his/her surrounding people like family, relatives, friends, and colleagues [56]. Their opinions sound important and valuable [30], and act as a motivational force suggesting one to avoid being excluded from the group or community by adjusting behavior to accord with social referents [21,53]. This viewpoint is consistent with the cognitive dissonance theory which believes that the conflict between an individual’s beliefs and behavior motivates him/her to alter attitude or action [57]. The association between subjective norms and behavioral intentions has been well documented in the tourism literature, for instance, positive connections were recorded between social norms and customer purchase intention on energy-efficient home appliances [58] and low-carbon footprint products at restaurants [59]. Hence, the second hypothesis was followed:

**H2.**  *Subjective norms positively impact tourist resource-saving behavioral intentions.*

The third element is perceived behavioral control which is regarded as ease or difficulty perception of a person’s concerning performing a behavior [22]. Perceived behavioral control shows a person’s estimation of the resources presented as opportunities or obstacles to adopt a given behavior [60]. If one perceives a high level of control over the behavior, this perception will encourage his/her engagement in that behavior [61]; otherwise, the perceived difficulty may discourage one from further action [53]. The findings of prior research confirmed a positive link between perceived behavioral control and intentions [62], for instance, consumers’ perceived behavioral control positively influenced their intentions to bring a reusable bag for shopping [63] and purchase green food [64]. The third hypothesis was thus put forward:

**H3.**  *Perceived behavioral control positively impacts tourist resource-saving behavioral intentions.*

Although TPB’s significant efficacy in examining various behaviors has been validated [65], it is incapable in all situations, and proper extensions would enhance its effectiveness [25]. The inclusion of environmental concern is believed to better fit the extended TPB theoretical framework in rural tourism context.

### 2.3. Environmental Concern (EC)

Environmental Concern (EC) is defined as the degree to which people are aware of environmental issues as well as support efforts/willingness to solve them [66]. It is used interchangeably with the term New Ecological Paradigm (NEP) in literature [67]. Compared with attitudes toward the behavior which indicate emotional evaluation of specific objects, environmental concern relates to an individual’s more general perceptions about the environment, quite often with unfavorable ones like worry, displeasure, uncertainty, and compassion [33,68,69].

Extant studies have demonstrated that environmental concern significantly influences behavioral intentions [70]. For instance, environmental concern significantly influenced consumers’ purchase intention for green products [71] as well as their green hotel visit intention [68]. Accordingly, the fourth hypothesis was suggested:

**H4.**  *Environmental concern positively impacts tourist resource-saving behavioral intentions.*

Environmental concern is also an important factor in driving environmental attitudes [28]. An individual with higher level of environmental concern tends to have a more positive perception of a product [72]. For instance, a positive link was observed between environmental concern and user’s attitude toward bicycle-sharing adoption [34]. Environmental concern also related positively to attitude towards eco-friendly apparel [73]. To this end, the research proposed the fifth hypothesis:

**H5.**  *Environmental concern positively impacts attitudes toward the behavior.*

An individual’s subjective norms are influenced via family and peer groups acting as “significant others” [70]. If one is highly environmentally concerned, he/she will obtain stronger support from reference people and show more interest in adopting hybrid electric vehicles [51] and accepting green energy brands [74]. Hence, the sixth hypothesis was drawn:

**H6.**  *Environmental concern positively impacts subjective norms.*

Having known the benefits of green consumption, an individual cares more about environment issues. Increased environmental concern reduces individual’s perception of difficulty when performing pro-environmental behaviors [70]. This was confirmed by positive connections between consumer’s environmental concern and perceived behavioral control on energy-efficient home appliances [75]. Thus, the last hypothesis was suggested:

**H7.**  *Environmental concern positively impacts perceived behavioral control.*

### 2.4. The Conceptual Model

**Figure 1:**
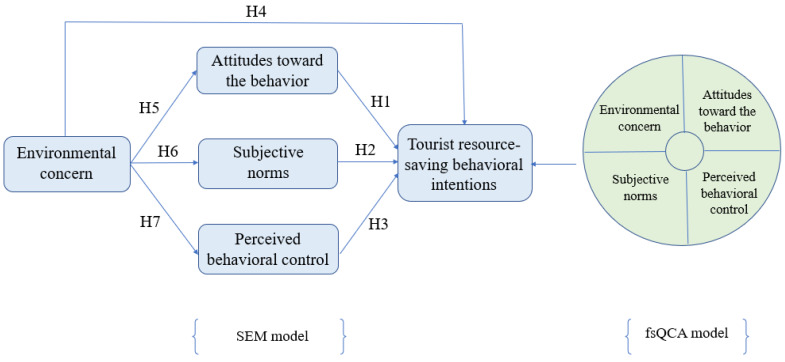
Conceptual model.

Based on the arguments above, this research presented the conceptual model (Figure 1) to explore tourist resource-saving behavioral intentions. This conceptual model is developed on the basis of the theory of the planned behavior originally proposed by Ajzen [22].

## 3. Method

### 3.1. Measurement

Construct measurements are listed in Table 1 by adopting 5-point Likert scales from 5 (“strongly agree”) to 1 (“strongly disagree”). All the items have been well identified, and some modifications were made to suit the specific situation. Appendix A presents detailed measurements of all constructs.

### 3.2. Pretest

Bilingual translation and back-translation were used for the survey. After that, a pretest was conducted before the formal survey. A panel of professionals including four destination practitioners and three tourism scholars were invited to evaluate the content validity [81]. Later, a sample of 80 qualified domestic tourists performed the pretest. While doing the questionnaire, they carefully checked all the items and gave feedback. The scale’s reliability and validity were both desirable [82].

### 3.3. Sample and Data Collection

The snowball sampling approach was selected and the reasons were twofold. First, this method has enjoyed a wide application in tourism research including tourist behavior [35]. Second, due to occasional occurrences of COVID-19 pandemic and strict preventive regulations across the country, on-site data collection turned out to be much more difficult. Therefore, the snowball sampling became a choice in recent research [83].

The procedure of the snowball sampling was conducted in this way: within their social network, the researchers sent the questionnaire to invited informants who then referred other informants to join the survey. The process repeated these steps until enough valid data has been collected [84]. Notably, informants as respondents in the snowball sampling methodology were selected with the following requirements: had visited a rural destination within one month, and had a clear memory of it [23]. Moreover, to achieve a successful survey, all research assistants were well trained about the sampling method.

The research team first found 9 qualified respondents (families, friends, relatives, and colleagues included) in their social network to complete the questionnaire. Then, each respondent invited 9 more respondents at most for the second-round questionnaire completion. This step was repeated until the final round. At last, a total of 600 questionnaires strictly adhering to requirements and steps were collected. A total of 546 questionnaires were valid, suggesting a 91% response rate. The sample presented a comparatively balanced gender ratio of 48.2% males and 51.8% females. Some 54% of the participants were aged below 25, and 46% were 25 years and older. Some 3.8% of them had middle school education or below; 16.7% were graduates of high school or technical secondary school; a large proportion of 79.5% received a Bachelor’s degree or above. With regard to the locations of rural tourism destinations that had been visited by participants, within the city accounted for 62.8%; not in the city but within the province, 25.8%; and outside the province, 11.4%. Among these rural destinations, many were at Yuhang District and Chun’an County of Hangzhou City, as well as Anji County of Huzhou City within Zhejiang Province. Twenty-one items corresponded to 546 valid responses in the questionnaire, suggesting the latter was over twenty times the former, which met Jackson (2003)’s requirement as an effective sample [85]. Both values of skewness (−0.839~−0.209) and kurtosis (−0.436~0.808) met the requirements as well [12].

### 3.4. Analysis Methods

This research applied both Structural equation modeling (SEM) and fuzzy-set qualitative comparative analysis (fsQCA) methods. The former as a variable-oriented approach analyzes linearity between variables, focusing on the net effect; the latter deals with asymmetric relationship between variables, offering insights into the non-linear effect, i.e., configurations of many variables for an outcome [86]. Hence, SEM was employed in this study to analyze linear associations between environmental concern, attitudes, subjective norms, perceived behavioral control, and tourist resource-saving behavioral intentions, while fsQCA was introduced to test combinational factors leading to resource-saving behavioral intentions.

## 4. Results

### 4.1. Measurement Model Assessment

As per Gerbing and Anderson (1988) [87], a two-step approach was adopted by examining the measurement model first and then the structural model [88]. The confirmatory factor analysis (CFA) tested the measurement before hypotheses assessment. Based on the conceptual model (see Figure 1), an overall goodness of fit test was performed on the measurement model. The results were: χ^2^/df = 1.955, RMR = 0.019, RMSEA = 0.042, GFI = 0.942, TLI = 0.978, NFI = 0.963, IFI = 0.981, CFI = 0.981, indicating a good fit to meet the requirement [89].

The overall measurement reliability was 0.947, with Cronbach’s α ranging from 0.886 to 0.942 as well as composite reliability (CR) 0.888 to 0.943, suggesting a good reliability of the measurement. Standardized factor loading of all constructs were between 0.716 to 0.916, a corresponding t-value 16.593 to 33.723, composite reliability 0.888 to 0.943, and average variance extracted (AVE) values 0.614 to 0.804, which also suggested a sufficient convergent validity. Additionally, discriminant validity was satisfied with the result that the 95% confidence interval of each pair of variables were exclusive of 1.0. Table 2 presents detailed results of the measurement model.

### 4.2. Structural Model Assessment

Structural equation modeling (SEM) was employed in the hypotheses relationship test. An overall goodness of fit test was performed on the structural model based on the conceptual model (see Figure 1). The results were: χ^2^/df = 3.484, RMR = 0.080, RMSEA = 0.068, GFI = 0.896, TLI = 0.943, NFI = 0.932, IFI = 0.951, CFI = 0.951. Though GFI was a bit lower than 0.9, the rest met requirements, which indicated an acceptable fit.

**Figure 2:**
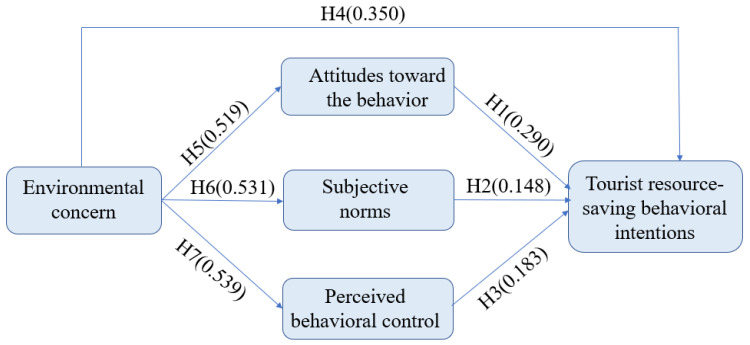
AMOS output results.

This study used the maximum likelihood method to exam the conceptual model (see Figure 1) and found all the hypothesis from H1-H7 were supported. Table 3 demonstrates detailed results and Figure 2 shows AMOS output result below.

### 4.3. Mediating Effect Examination

The bootstrapping method was performed to examine the mediating effects. Results from Table 4 prove that the mediating effect of attitudes was identified on the relationship between environmental concern and tourist resource-saving behavioral intentions (CI _Attitudes_ = [0.0777, 0.1525]), supporting the environmental concern→attitudes→tourist resource-saving behavioral intentions path. Likewise, given the confidence interval does not include 0, mediation roles of subjective norms and perceived behavioral control were also confirmed respectively through linkages between environmental concern and resource-saving behavioral intentions (CI _Subjective norms_ = [0.0325, 0.1066]; CI _perceived behavioral control_ = [0.0335, 0.1080]). Furthermore, there were no significant differences between the three mediation paths aforementioned (CI _C1-C2_ = [−0.0049, 0.1043]; CI _C1-C3_ = [−0.0026, 0.0959]; CI _C2-C3_ = [−0.0634, 0.0586]).

Also from Table 4, regarding the influential effect of environmental concern on tourist resource-saving behavioral intentions, the total indirect effect took 46.76% of total effect, among which the specific mediating path of environmental concern→attitudes→tourist resource-saving behavioral intentions accounted for 21.50% of the total effect; environmental concern→subjective norms→tourist resource-saving behavioral intentions, 12.39%; and environmental concern→perceived behavioral control→tourist resource-saving behavioral intentions, 12.87%.

### 4.4. FsQCA Assessment

#### 4.4.1. Contrarian Case Analysis

Ahead of fuzzy-set qualitative comparative analysis (fsQCA), contrarian case analysis was performed to effectively examine how many instances in the collected sample have not been explained by the main effects. They would not thereby be included in the output of a normal variance-based method [90]. As some scholars emphasized, when doing variable-level analysis, researchers’ common mistake is their ignorance of cases of association opposing to the main effect relationship [91]. Accordingly, contrarian case analysis is suggested to identify possible positive, negative, or non-existing associations in the same dataset [92].

As per the advice of Pappas and Woodside (2021) on contrarian case analysis [93], the sample of the research was divided first by quintiles in order to check the relationships between variables. The quintiles were then tested by cross-contingency analysis. Any two variables’ cross-contingency analysis outcome is a 5 × 5 table, presenting the two variables’ every possible configuration at each quantile in the sample. Cases at the upper left and lower right corners of the table signify main effects, while cases at the rest two opposite corners (i.e., the lower left and upper right) are on the contrary, unable to be explained by main effects. The existent cases at the lower left and upper right corners indicate the existence of contrarian cases in the sample. Table 5, Table 6, Table 7 and Table 8 record the cross-contingency of attitudes, subjective norms, perceived behavioral control, environmental concern, and tourist resource-saving behavioral intentions. All these tables demonstrate contrarian cases in the sample. As such, fsQCA was introduced to incorporate counterfactual cases in exploring high-level resource-saving behavioral intentions.

#### 4.4.2. Data Calibration

The outcome (i.e., tourist resource-saving behavioral intentions) and every condition (i.e., attitudes toward the behavior, subjective norms, perceived behavioral control, environmental concern) in fsQCA is regarded as a separate set. Every case has a member score in the set. The data calibration, therefore, is the process of assigning a set member value to each case [94]. When a variable is measured by multiple items, each case in the variable needs an assigned value as an fsQCA input value. The simplest way is to average all items and input a single value correspondingly for each case [93]. Following Moreno et al. (2016) [95], each variable’s calibration standard for full non-member was set to 0.05th percentile; for the intersection, 0.5th percentile; and for full member, 0.95 percentile. Table 9 records each condition and outcome’s calibration information.

#### 4.4.3. fsQCA Necessary Condition Assessment

In line with Crilly, Zollo, and Hansen (2012)’s criteria [96], This study adjusted input values of 0.5 after calibration to 0.499. It is required to check the necessity of each condition individually before the conditional configuration analysis starts [94]. The fsQCA software was employed to verify whether a single condition (its non-set included) constitutes a necessary condition for tourist resource-saving behavioral intentions. During QCA assessment, if a particular condition exists till the result appears, this condition is counted as a necessary one for the result [97]. Additionally, given consistency as an important standard of a necessary condition, if a consistency value is higher than 0.9, this condition is deemed as necessary condition for the result [97]. Table 10 shows necessary condition results of high- and non-high-level tourist resource-saving behavioral intentions, in which the consistency of all conditions was lower than 0.9. It can be concluded that no necessary condition was there for affecting high- and non-high-level tourist resource-saving behavioral intentions.

#### 4.4.4. Sufficiency Assessment of Configuration Conditions

As per Fiss (2011) [98] and Du and Jia (2017) [99], the research set 0.8 as the consistency threshold; 0.7, the PRI consistency threshold; 2, the case frequency threshold; and retained leastwise 80% of the sample. Each variable’s outcomes after the configuration analysis process are listed in Table 11. As to the three configurations in this table, consistency level whether of the single solution (configuration) or overall solution was above 0.75, the acceptable minimum standard [94], of which the overall solution’s consistency was 0.883, and the overall solution’s coverage, 0.700; thereby the three configurations in the table are verified as a sufficient condition combination for high-level tourist resource-saving behavioral intentions.

The antecedent configuration of tourist resource-saving behavioral intentions is categorized into the TPB mode (configuration 1 in the Table 11) and dual-attitudes mode (configuration 2 and configuration 3 in the Table 11).

**Figure 3:**
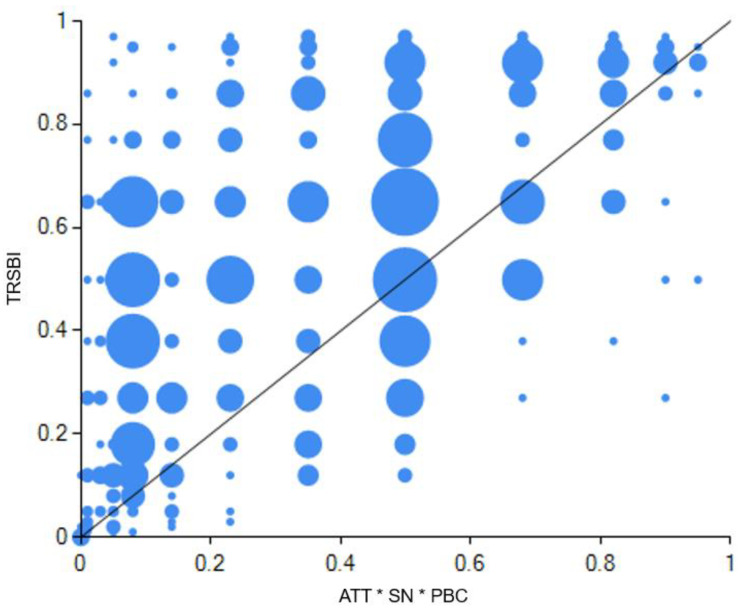
Configuration 1 explanation in the mode of tourist resource-saving behavioral intentions.

The three traditional variables of TPB in configuration 1 together play a core role, which suggests that when attitudes, subjective norms and perceived behavioral control coexist, rest conditions are not relevant for high-level tourist resource-saving behavioral intentions. In other words, compared with rest conditions, the traditional TPB framework is particularly critical for tourist resource-saving behavioral intentions, as this framework alone constitutes a sufficient condition for outcomes. Therefore, this configuration is named as TPB mode in this study. Its consistency was 0.906; the unique coverage, 0.052; and the raw coverage, 0.595. This path explained some 59.45% of the cases of tourist resource-saving behavioral intentions. Figure 3 demonstrates a detailed explanation of configuration 1.

**Figure 4:**
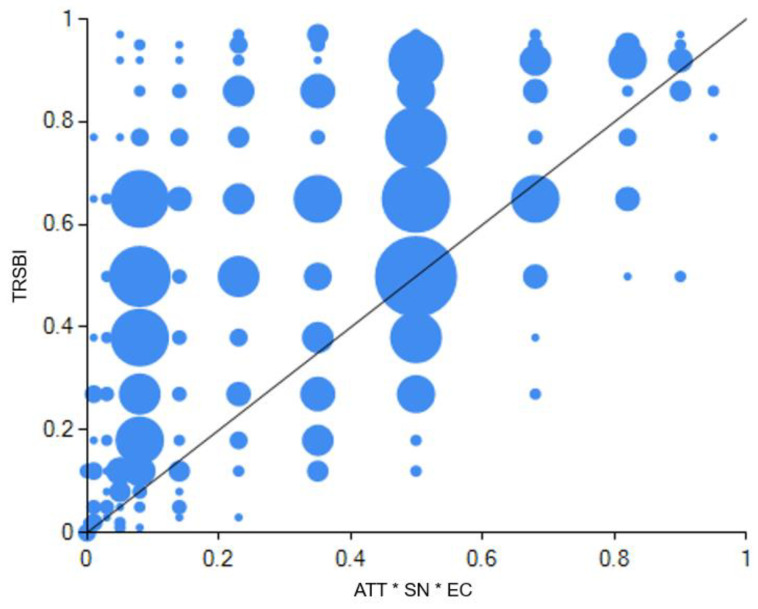
Configuration 2 explanation in the mode of tourist resource-saving behavioral intentions.

**Figure 5:**
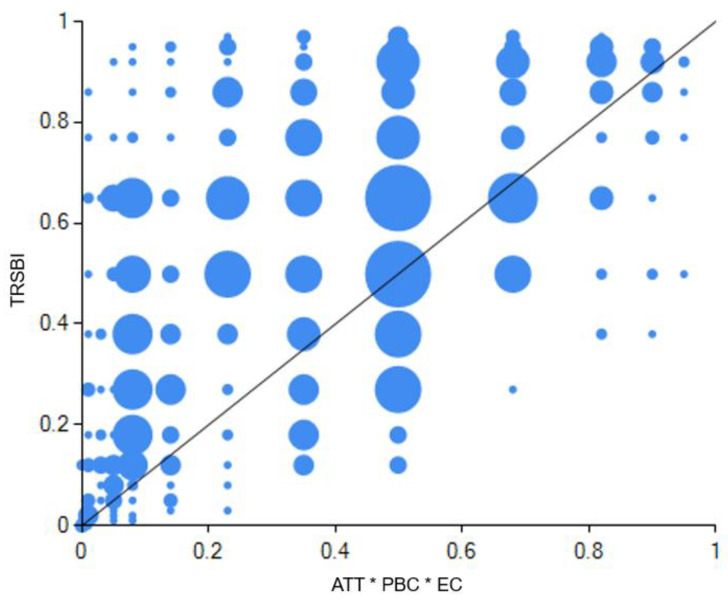
Configuration 3 explanation in the mode of tourist resource-saving behavioral intentions.

In the dual-attitudes mode, the core condition was attitudes and environmental concern, which mainly consist of two sub-modes (i.e., configuration 2 and configuration 3). This means that compared with other conditions, the coexistence of environmental concern at a general level and attitudes toward the behavior in a specific context was particularly important for tourist resource-saving behavioral intentions. Figure 4 and Figure 5 demonstrate detailed explanations of configuration 2 and configuration 3.

#### 4.4.5. Robustness Testing

The robustness test was conducted by adjusting the consistency threshold level from 0.8 to 0.85, and no substantial changes were found in the configuration number, configuration components, or consistency and coverage’s fitting parameters. The current study’s reliability was enhanced [100].

### 4.5. Findings

The findings of SEM approach were: first, attitudes toward the behavior, subjective norms, perceived behavioral control and environmental concern all had significant and direct effects on tourist resource-saving behavioral intentions; second, environmental concern significantly and directly impacted attitudes, subjective norms, and perceived behavioral control; third, the three TPB endogenous constructs (i.e., attitudes toward the behavior, subjective norms, and perceived behavioral control) all played significant mediating roles in the relationship between environmental concern and tourist resource-saving behavioral intentions; fourth, among the three endogenous TPB variables, attitudes not only is the most important factor in driving tourist resource-saving behavioral intentions, but also the most important mediation construct in the relationship between environmental concern and tourist resource-saving behavioral intentions.

The findings of the fsQCA approach were: first, none of the four variables (i.e., attitudes toward the behavior, subjective norms, perceived behavioral control, and environmental concern) could alone form a sufficient and necessary condition in exploring tourist resource-saving behavioral intentions; second, the aforementioned four variables generated sixteen condition combinations, among which three configurations met the requirements; the overall coverage rate was 0.700. Specifically, the three configurations resulting in a high level of TRSBI were: (1) high attitudes toward the behavior, subjective norms, and perceived behavioral control, (2) high attitudes toward the behavior, subjective norms, and environmental concern, (3) high attitudes toward the behavior, perceived behavioral control, and environmental concern. Hence, two modes, i.e., the TPB and dual-attitudes modes were constituted.

## 5. Conclusions, Contributions, and Implications

### 5.1. Conclusions

Tourist resource-saving behavioral intentions bring great significance to rural destinations’ sustainability. A conceptual model is developed based on TPB and we assessed the formation mechanism and configuration effects of tourist resource-saving behavioral intentions in rural tourism context through a combined approaches of Structural equation modeling (SEM) and fuzzy-set qualitative comparative analysis (fsQCA).

In line with prior studies [29,34], SEM findings confirmed the positive significant influence of environmental concern on TPB endogenous constructs (i.e., attitudes toward the behavior, subjective norms, and perceived behavioral control), as well as the three constructs’ mediating effects in the relationship between environmental concern and tourist resource-saving behavioral intentions, which supported the argument that environmental concern is an important antecedents of tourist resource-saving behavioral intentions in the TPB framework by both direct and indirect influences [73].

In the light of previous research [35], fsQCA findings implied that this approach is useful in complementing SEM and explaining the complexity of behavioral outcomes. Drawn from this study’s results, their commonalities lie as follows: first, both methods highlighted the importance of TPB endogenous constructs in predicting resource-saving behavioral intentions. Second, attitudes as a vital variable exerted both direct and mediating effects in this relationship, confirming attitudes’ vigorous influence on behavioral intentions [29]. fsQCA outcomes revealed that the three paths driving resource-saving behavioral intentions could be converted into two modes. Whether it be TPB or dual-attitudes mode, attitudes are one of the essential conditions. However, environmental concern as an independent determinant of resource-saving behavioral intentions was verified only by SEM analysis, and not by fsQCA analysis. In detail, fsQCA outcomes showed that environmental concern was not a sufficient and necessary condition in predicting resource-saving behavioral intentions, not constituting a configuration. In this sense, combining the two methods is crucial in exploring both linear and non-linear relationships among factors leading to resource-saving behavioral intentions.

### 5.2. Theoretical Contributions

The current study used the theory of the planned behavior (TPB) to explore the formation mechanism of tourist resource-saving behavioral intentions in rural tourism context. The extant literature has made substantial efforts in applying TPB into tourism research and demonstrated its robustness [23]. Unfortunately, much less research to date has been made targeting rural tourist resource-saving behavioral intentions [17]. This study filled the gap with empirical results, verifying TPB’s significance in explaining individual decision-making process [22], and meanwhile extending its academic value to the field of resource-saving behavioral intentions.

Second, this research integrated environmental concern into TPB framework and discussed its mechanism for influencing tourist resource-saving behavioral intentions. Research on the significance and scale development of environmental concern is not new [66], neither is the investigations as an important determinant of ecological behavioral decisions [33]; however, attention to its influence mechanism on resource-saving behavioral intentions is very recent. Environmental concern worked as the source factor in driving resource-saving behavioral intentions, which proved its prominent role in environmental issues [28], again corroborated the effectiveness of extended TPB in predicting individual decision making [22], and enriched its domain with resource-saving research.

Third, this study combined SEM and fsQCA to identify antecedents of tourist resource-saving behavioral intentions. The extant literature in tourism research mainly employed SEM to test linear connections between variables, while fsQCA was rarely utilized in this area [35]. Remarkably, tourist resource-saving behavioral intentions as segment pro-environmental behavioral intentions, is a complex phenomenon triggered by multiple concurrent causes and effects. The SEM method validated linear associations between variables, while the fsQCA approach corroborated multiple antecedent combination patterns to form tourist resource-saving behavioral intentions. The combination of SEM and fsQCA in this study could provide evidence for this methodological integration [101] and extend the application in rural tourism contexts [35].

### 5.3. Practical Implications

Empirical findings of the current research underscore the TPB and dual-attitudes modes in shaping tourist resource-saving behavioral intentions, which provides practical implications for sustainability of rural destinations.

First, both SEM and fsQCA results demonstrated that whether linearly or configurationally, TPB constructs are powerful predictors of tourist resource-saving behavioral intentions. As such, there is still room for improving attitudes, subjective norms, and perceived behavioral control, and more importantly strengthening their synergistic integration to achieve the targeted behavioral outcomes. Rural destination management organizations (DMO) should endeavor to match multiple conditions among the aforementioned three variables and formulate measures with a holistic perspective to promote tourist resource-saving behavioral intentions. Specifically, as supported by empirical results, it is paramount to nurture positive attitudes toward resource saving. Destination management departments should reinforce tourist perception of their ability and contribution through encouraging them to learn the significance of conserving resources for destination sustainability. Moreover, different stakeholders should make joint efforts to motivate tourists. Local government as well as DMO must take the lead in publicity both online and offline. Especially when online, special attention should be paid when dealing with remarks on social media [102]. It is always a good way to respond efficiently and politely [103]. Moreover, reminding roles of destination employees, residents, family-based small tourism business and tour guides should never be overlooked, as they have direct interaction with tourists [104,105,106,107]. Particularly, with residents who quite often offer homestay and spend longer time with tourists, their function in propelling tourist to save resources is pivotal and significant [108]. DMO should inform tourists of potential violation and fines for lavishment [109]. Furthermore, they should always consider how to make it easier for tourists to engage in resource-saving. Apart from infrastructure investment, improving extant facilities to be more user-friendly is worth exploring. Recognition, awards, and discount could also be considered as diverse incentive means [110]. All rural destination stakeholders must cooperate well so as to foster tourist resource-saving behavioral intentions.

Second, regarding dual-attitudes’ momentous roles, rural tourism destinations should enhance the synergistic integration of the two attitude factors, i.e., environmental concern and attitudes. Stressing attitudes alone cannot fully guarantee the generation of resource-saving behavioral intentions at rural destinations. Moreover, albeit specific attitudes could be activated in certain rural tourism scenarios, the cultivation of environmental concern as general attitudes is a systematic and long-run course. As such, paying attention to both environmental concern and attitudes can nurture tourist resource-saving behavioral intentions. Given the above consideration, on the one hand, to provoke specific attitudes at rural destination, tourists themselves and all stakeholders at destination should play their own roles well as suggested ahead [111]; on the other hand, destination managers may be able to cultivate environmental concern by integrating resource-saving into a destination’s long-run development strategy. They could launch constant campaigns focusing on environmental protection through individual awareness and contribution, urging people to accept resource-saving as a social norm. People who have a deep awareness of or interest in environmental protection are more willing to engage in environmental issues [112]. For instance, online events on how to save resources via destination official social network account is a great promoter of tourists’ knowledge acquisition, their active interaction with the destination, and their bonding with the destination [113]. When undertaking promotion, it is a good practice to invite key opinion leaders to participate and set good examples for the public. Besides establishing pro-environmental reputation and winning public trust [114], DMOs can consider introducing smart technology into conspicuous places at the rural destination and tourist accommodation [115], which, with constant monitoring and synchronous analysis, might give tourists a better understanding of destination environment at the present, stimulate their environmental consciousness, and guide them to be active in performing resource-saving actions.

## 6. Limitations and Future Research

First, the snowball sampling method in this study leaves room for further discussion. It was used in some studies recently [116,117] especially due to frequent outbreaks of the COVID-19 pandemic, and was indeed a popular option when on-site survey became more inconvenient to conduct. Future research could adopt other sampling approaches if the pandemic eases, and experimental research can also be taken into account [118,119]. Second, the theoretical framework could be further enriched based on literature, for instance, by introducing emotional determinants [120,121,122,123] and enhancing demographic features [124] to test the robustness of the integrated model. Third, more segment tourist resource saving behaviors [125], for instance, food waste reduction [126] particularly plate waste reduction [127] and waste sorting [62], can be explored to further the research.

## Figures and Tables

**Table 1:** The measurement items.

Construct	Item	Source
Attitudes toward the behavior (ATT)	ATT1	Song et al. (2012) [76]
	ATT2	
	ATT3	
	ATT4	
Subjective norms (SN)	SN1	Lee et al. (2012); Song et al. (2014) [77,78]
	SN2	
	SN3	
	SN4	
Perceived behavioral control (PBC)	PBC1	Meng & Choi (2016) [30]
	PBC2	
	PBC3	
	PBC4	
Environmental concern (EC)	EC1	Han (2015); Kiatkawsin & Han (2017) [65,79]
	EC2	
	EC3	
	EC4	
Tourist resource-saving behavioral intentions (TRSBI)	TRSBI1	Qiu & Zhou (2017) [80]
	TRSBI2	
	TRSBI3	
	TRSBI4	
	TRSBI5	

**Table 2:** Results of the measurement model.

Construct	Loading	t-Values	Reliability	CR	AVE
SN1	0.828	25.382	0.926	0.926	0.758
SN2	0.889	29.044			
SN3	0.887	28.905			
SN4	0.877	—			
ATT1	0.864	29.476	0.942	0.943	0.804
ATT2	0.906	32.887			
ATT3	0.916	33.723			
ATT4	0.900	—			
PBC1	0.815	21.082	0.899	0.899	0.691
PBC2	0.855	22.427			
PBC3	0.852	22.311			
PBC4	0.801	—			
EC1	0.826	22.601	0.913	0.914	0.727
EC2	0.886	25.006			
EC3	0.875	24.551			
EC4	0.822	—			
TRSBI 1	0.825	18.319	0.886	0.888	0.614
TRSBI 2	0.861	19.060			
TRSBI 3	0.746	16.593			
TRSBI 4	0.762	16.950			
TRSBI 5	0.716	—			

Note: SN, subjective norms; ATT, attitudes toward the behavior; PBC, perceived behavioral control; EC, environmental concerns; TRSBI, tourist resource-saving behavioral intentions; CR, composite reliability; AVE, average variance extracted.

**Table 3:** Structural model assessment and hypothesis examination result.

Hypothesis & Path	Standardized Coefficient	t-Value	Result
H1: ATT→TRSBI	0.290 ***	6.781	Supported
H2: SN→TRSBI	0.148 ***	3.529	Supported
H3: PBC→TRSBI	0.183 ***	4.196	Supported
H4: EC→TRSBI	0.350 ***	6.117	Supported
H5: EC→ATT	0.519 ***	11.760	Supported
H6: EC→SN	0.531 ***	11.813	Supported
H7: EC→PBC	0.539 ***	11.432	Supported

Note: * *p* < 0.05; ** *p* < 0.01; *** *p* < 0.001. SN, subjective norms; ATT, attitudes toward the behavior; PBC, perceived behavioral control; EC, environmental concerns; TRSBI, tourist resource-saving behavioral intentions.

**Table 4:** Specific mediation analysis results.

Types of Indirect Mediating Path	Effect Size	Boot SE	Boot LLCI	BootULCI	Account for Total Effect
Total indirect effect	0.2441	0.0292	0.1894	0.3049	46.76%
Specific mediating paths					
EC→ATT→TRSBI(C1)	0.1123	0.0190	0.0777	0.1525	21.50%
EC→SN→TRSBI(C2)	0.0647	0.0187	0.0325	0.1066	12.39%
EC→PBC→TRSBI(C3)	0.0672	0.0191	0.0335	0.1080	12.87%
Significant differences between different specific mediating paths					
C1—C2	0.0476	0.0276	−0.0049	0.1043	—
C1—C3	0.0451	0.0254	−0.0026	0.0959	—
C2—C3	−0.0025	0.0310	−0.0634	0.0586	—

Note: SN, subjective norms; ATT, attitudes toward the behavior; PBC, perceived behavioral control; EC, environmental concerns; TRSBI, tourist resource-saving behavioral intentions.

**Table 5:** Cross-contingency of attitudes and tourist resource-saving behavioral intentions.

Attitudes toward the Behavior		Tourist Resource-Saving Behavioral Intentions					Total
Cramer’s V = 0.302, *p* < 0.001		1	2	3	4	5	
1	Case number	56	23	22	**8**	**2**	111
	Percentage	50.5%	20.7%	19.8%	**7.2%**	**1.8%**	100.0%
2	Case number	24	14	18	**8**	**3**	67
	Percentage	35.8%	20.9%	26.9%	**11.9%**	**4.5%**	100.0%
3	Case number	23	42	50	18	15	148
	Percentage	15.5%	28.4%	33.8%	12.2%	10.1%	100.0%
4	Case number	**4**	**12**	40	18	26	100
	Percentage	**4.0%**	**12.0%**	40.0%	18.0%	26.0%	100.0%
5	Case number	**4**	**6**	32	38	40	120
	Percentage	**3.3%**	**5.0%**	26.7%	31.7%	33.3%	100.0%
Total	Case number	111	97	162	90	86	546
	Percentage	20.3%	17.8%	29.7%	16.5%	15.8%	100.0%

**Table 6:** Cross-contingency of subjective norms and tourist resource-saving behavioral intentions.

Subjective Norms		Tourist Resource-Saving Behavioral Intentions					Total
Cramer’s V = 0.280, *p* < 0.001		1	2	3	4	5	
1	Case number	51	18	24	**4**	**3**	100
	Percentage	51.0%	18.0%	24.0%	**4.0%**	**3.0%**	100.0%
2	Case number	37	30	37	**18**	**9**	131
	Percentage	28.2%	22.9%	28.2%	**13.7%**	**6.9%**	100.0%
3	Case number	18	26	43	30	15	132
	Percentage	13.6%	19.7%	32.6%	22.7%	11.4%	100.0%
4	Case number	**1**	**7**	27	10	10	55
	Percentage	**1.8%**	**12.7%**	49.1%	18.2%	18.2%	100.0%
5	Case number	**4**	**16**	31	28	49	128
	Percentage	**3.1%**	**12.5%**	24.2%	21.9%	38.3%	100.0%
Total	Case number	111	97	162	90	86	546
	Percentage	20.3%	17.8%	29.7%	16.5%	15.8%	100.0%

**Table 7:** Cross-contingency of perceived behavioral control and tourist resource-saving behavioral intentions.

Perceived Behavioral Control		Tourist Resource-Saving Behavioral Intentions					Total
Cramer’s V = 0.282, *p* < 0.001		1	2	3	4	5	
1	Case number	46	29	20	**9**	**7**	111
	Percentage	41.4%	26.1%	18.0%	**8.1%**	**6.3%**	100.0%
2	Case number	18	16	12	**12**	**9**	67
	Percentage	26.9%	23.9%	17.9%	**17.9%**	**13.4%**	100.0%
3	Case number	21	41	36	26	24	148
	Percentage	14.2%	27.7%	24.3%	17.6%	16.2%	100.0%
4	Case number	**3**	**12**	12	38	35	100
	Percentage	**3.0%**	**12.0%**	12.0%	38.0%	35.0%	100.0%
5	Case number	**4**	**10**	11	47	48	120
	Percentage	**3.3%**	**8.3%**	9.2%	39.2%	40.0%	100.0%
Total	Case number	92	108	91	132	123	546
	Percentage	16.8%	19.8%	16.7%	24.2%	22.5%	100.0%

**Table 8:** Cross-contingency of environmental concern and tourist resource-saving behavioral intentions.

Environmental Concern		Tourist Resource-Saving Behavioral Intentions					Total
Cramer’s V = 0.221, *p* < 0.001		1	2	3	4	5	
1	Case number	50	13	31	**11**	**6**	111
	Percentage	45.0%	11.7%	27.9%	**9.9%**	**5.4%**	100.0%
2	Case number	25	9	21	**10**	**2**	67
	Percentage	37.3%	13.4%	31.3%	**14.9%**	**3.0%**	100.0%
3	Case number	25	12	45	36	30	148
	Percentage	16.9%	8.1%	30.4%	24.3%	20.3%	100.0%
4	Case number	**7**	**14**	41	22	16	100
	Percentage	**7.0%**	**14.0%**	41.0%	22.0%	16.0%	100.0%
5	Case number	**12**	**5**	32	41	30	120
	Percentage	**10.0%**	**4.2%**	26.7%	34.2%	25.0%	100.0%
Total	Case number	119	53	170	120	84	546
	Percentage	21.8%	9.7%	31.1%	22.0%	15.4%	100.0%

**Table 9:** Condition and outcome calibration in the model of tourist resource-saving behavioral intentions.

Category	Condition and Outcome	Calibration		
		Full Member	Intersection	Full Non-Member
Outcome variable	Tourist resource-saving behavioral intentions	4.8	3.8	2.6
Condition variable	Attitudes toward the behavior	5.0	4.0	2.75
	Subjective norms	5.0	4.0	2.75
	Perceived behavioral control	4.75	3.75	2.50
	Environmental concern	5.0	4.0	2.75

**Table 10:** Necessary condition analysis in the model of tourist resource-saving behavioral intentions.

Condition Variable	Tourist Resource-Saving Behavioral Intentions		~Tourist Resource-Saving Behavioral Intentions	
	Consistency	Coverage	Consistency	Coverage
Attitudes toward the behavior	0.800	0.795	0.580	0.503
~Attitudes toward the behavior	0.500	0.578	0.764	0.769
Subjective norms	0.725	0.819	0.537	0.529
~Subjective norms	0.583	0.591	0.816	0.721
Perceived behavioral intentions	0.798	0.782	0.605	0.517
~Perceived behavioral intentions	0.507	0.596	0.745	0.763
Environmental concern	0.828	0.774	0.626	0.510
~Environmental concern	0.476	0.594	0.723	0.785

**Table 11:** Configuration assessment of high-level tourist resource-saving behavioral intentions.

Mode	TPB Mode	Dual-Attitudes Mode	
Condition Configuration	Configuration 1	Configuration 2	Configuration 3
Attitudes toward the behavior	●	●	●
Subjective norms	●	●	
Perceived behavioral control	●		●
Environmental concern		●	●
Consistency	0.906	0.916	0.899
Raw coverage	0.595	0.578	0.613
Unique coverage	0.052	0.035	0.070
Overall consistency		0.883	
Overall coverage		0.700	

Note: • or • denotes the existence of a condition, ⊗ or ⊗ denotes its absence; • or ⊗ indicates core condition, • or ⊗ indicates marginal condition. The blank represents “don’t care” condition.

## Data Availability

Not applicable.

## Appendix A. Detailed Measurements of All Constructs

**Construct**	**Item**	**Source**
Attitudes toward the behavior (ATT)	ATT1 I think saving resources at this rural destination is a positive behavior.	Song et al. (2012) [76]
	ATT2 I think saving resources at this rural destination is a valuable behavior.	
	ATT3 I think saving resources at this rural destination is a necessary behavior.	
	ATT4 I think saving resources at this rural destination is a beneficial behavior.	
Subjective norms (SN)	SN1 Most people who are important to me understand I should save resources at this rural destination.	Lee et al. (2012); Song et al. (2014) [77,78]
	SN2 Most people who are important to me support that I should save resources at this rural destination.	
	SN3 Most people who are important to me recommend that I save resources at this rural destination.	
	SN4 Most people who are important to me agree that I save resources at this rural destination.	
Perceived behavioral control (PBC)	PBC1 Whether or not I save resources at this rural destination is up to me.	Meng & Choi (2016) [30]
	PBC2 I am capable of saving resources at this rural destination.	
	PBC3 I am confident that if I want, I can save resources at this rural destination.	
	PBC4 I have enough resource, time and opportunities to save resources at this rural destination.	
Environmental concern (EC)	EC1 Humans are severely abusing the environment.	Han (2015); Kiatkawsin & Han (2017) [65,79]
	EC2 The earth is like a spaceship with limited room and resources.	
	EC3 If we continue to develop regardless of the environment, we will soon experience environmental catastrophe.	
	EC4 Human damage to nature often produces disastrous consequences.	
Tourist resource- saving behavioral intentions (TRSBI)	TRSBI1 I intend to save electricity at this rural destination.	Qiu & Zhou (2017) [80]
	TRSBI2 I intend to save water at this rural destination.	
	TRSBI3 I intend to walk on feet and use local transportation or non-motor vehicles at this rural destination.	
	TRSBI4 I intend to use recyclable things at this rural destination.	
	TRSBI5 I intend to reduce food waste at this rural destination.	
